# Navigating the energy transition: Identifying critical success factors for ancillary services provision and sustainable energy solutions in Germany

**DOI:** 10.1016/j.heliyon.2024.e27643

**Published:** 2024-03-06

**Authors:** Jana Gerlach, Vanessa Beutel, Carsten Wegkamp, Michael H. Breitner, Stefan Geißendörfer, Bernd Engel, Karsten von Maydell

**Affiliations:** a Leibniz Universität Hannover, Information Systems Institute, Königsworther Platz 1, 30167, Hannover, Germany; b German Aerospace Center, DLR Institute of Networked Energy Systems, Carl-von-Ossietzky-Straße 15, 26129, Oldenburg, Germany; c Technische Universität Braunschweig, Elenia Institute for High Voltage Technology and Power Systems, Schleinitzstraße 23, 38106, Braunschweig, Germany

**Keywords:** Ancillary services, Critical success factors, Transmission system operators, Plant operators, Distribution system operators

## Abstract

The provision of ancillary services (AS) is subject to changes associated with the energy transition. Due to new requirements, the power supply quality, reliability, and safety must be achieved by simultaneously complying with technological, economic, and environmental constraints. To mitigate these challenges, we derive factors responsible for a successful venture of all stakeholders, referred to as critical success factors (CSFs). In a Design Science Research (DSR)-based approach, twelve specific CSFs are deduced from expert interviews with transmission-, plant-, and distribution system operators. These CSFs are evaluated in a focus group discussion with academic experts afterward. We summarize practical results and findings from failed and successful projects concerning energy trading strategies, asset portfolios, grid expansion, and communication technologies. We contribute to AS knowledge and derive recommendations for further research and practice.

## 1 Introduction

Marked by decentralization, decarbonization, and digitalization, the energy sector undergoes a transformation that necessitates the integration of distributed generation plants, including photovoltaic (PV) systems, wind power plants (WPP) or biogas plants (BP). In addition, there is an electrification of the mobility and heating sector, which involves components like electric vehicles (EV) or heat pumps (HP) [[1], [2], [3]]. The Paris Climate Agreement, EU Green Deal, and national legislation drive the energy landscape of Europe, especially Germany [4,5]. However, this transition poses challenges to ensure a reliable and secure power supply with an increasing share of renewable energies [1]. AS, provided by transmission system operators (TSOs), plays a crucial role in maintaining grid stability amidst the changing energy generation landscape. Conventional AS from large central power plants must be replaced by distributed renewable generation systems, consumption components, and storage systems [6,7]. This shift places new demands on stakeholders, including TSOs, plant operators (POs), and distribution system operators (DSOs), who must ensure power supply quality, reliability, safety, and efficiency [1,2]. To address these challenges, technological innovations, market strategies, and information processes must evolve to ensure continued economic efficiency [2,8].

Current literature on future AS provision examines specific plants, such as battery storage systems (BSS) [9,10], virtual power plants (VPP) [11], and EVs in terms of their contribution to grid stabilization [12]. Researchers employ various methods, including mathematical models [11], simulations [13], and literature reviews [14]. For instance, Dong et al. [13] simulated the effects of decentralized power provision based on distributed energy resources (DER) on reliability and economic efficiency. Saldaña et al. [14] investigated EV charging strategies through a literature review, while Lymperopoulos et al. [12] conducted a techno-economic analysis, and Osório et al. [15] conducted a case study. The literature also explores the impact of smart microgrids on AS provision, with literature reviews by Martinez-Ramos et al. [16], simulations by Di Silvestre et al. [17], and case studies by Helguero et al. [18]. Additionally, the inclusion of energy end-consumers in smart buildings is investigated using artificial neural networks and mathematical modeling, as seen in studies by De Zotti et al. [19], Siano & Mohammad [20], and Avramidis et al. [21]. An aggregation of these technological and economic factors responsible for a successful future AS provision and the integration of practical experts participating in the energy sector is limited in existing research. According to Einolander and Kidviaho [22], further research on the AS environment requires interviews with practicing stakeholders and decision-makers. Therefore, investigating CSFs for a successful and competitive future provision of AS deduced from practical insights through expert interviews can address this research demand. This leads to the following research question (RQ):

RQ What are critical success factors for the future provision of ancillary services in the electrical power grid?

Per definition, CSFs aim to “list the main factors that distinguish between project failure and project success” [23, p. 3433]. We deduce CSFs for future AS provision to address our RQ by following the DSR approach as our research scheme, according to Gregor and Hevner [23] and vom Brocke et al. [24]. By interviewing TSOs, DSOs, and POs, we gain practical insights to deduce specific CSFs related to technical and economic aspects of AS provision. First, we provide theoretical foundations, describe our research design, and derive our CSFs. Then, we discuss our results and findings and highlight our implications and recommendations for practice and further research. In addition, we identify linkages between specific CSFs and visualize them in a dodecahedron. Our CSFs contribute to theory and practice by providing practical insights on economic benchmarks in AS pricing, distribution channels, competition, technical requirements, electrical capabilities, and future strategic directions.

## 2 Theoretical background

The power system consists of all installations needed to generate, transport, and consume electrical energy [25]. The grid is all interconnected components, equipment, and installations. It features a hierarchical structure using several grids with different nominal voltages connected via transformers between the voltage levels [26]. European industrialized countries have four grid levels: The transmission grid represents the extra-high voltage level and is responsible for long-range power transport. The distribution grid consists of the high voltage (HV) level, the medium voltage (MV) level, and the low voltage (LV) level [27].

In conventional grids, the electric power was generated at higher voltage levels, transmitted over long ranges, and distributed in smaller areas to demand facilities like industry or households [28]. Due to the major changes in the implementation, the historically evolved grid structure is put into situations with a higher stress level [29]. As a result, to ensure a reliable grid operation within the permitted limits of the grid equipment, one option is to conventionally expand the grid in the form of bigger and more lines [1].

In addition to very high investments, different stakeholders have other concerns [30]. On the one hand, TSOs and DSOs aim to ensure that the reliability of grid operation is most easily achieved through grid expansion. On the other hand, the population wants a low environmental impact and has to pay for grid expansion investments through grid fees [31]. Thus, another possibility is the utilization of installed plants for grid operation, which introduces POs as another stakeholder. These can be single prosumer households with a PV and BSS, small public utilities, or large corporations with a huge asset portfolio. Their main target is profit maximization with a modest impact on their equipment [32]. Finally, there are many stakeholders when it comes to the problem of changing the provision of AS for reliable grid operation in the future energy system. For this reason, interviews with different stakeholders are of high importance.

Regarding the future AS contribution, the interviews focus on AS for frequency and voltage stability [7]. First, voltage stability is a non-frequency-related system service. The interviews focus on static voltage control, which refers to providing reactive power by a generating unit or grid component (e.g., regulated substations or compensation plants) to balance voltage during normal operation [33]. Static voltage control is intended to keep the distribution grid in a steady-state voltage fluctuation within permitted limits [7]. Causes for static voltage fluctuations can be changes in load, generation, or grid conditions [34].

Frequency stability describes the balance between electricity generation and consumption at any point in time. Besides other factors, such as inertia, the main product within frequency stability is the provision of control reserve (CR). These services are staggered CRs that are traded market-based [35]. The CR includes the Frequency Containment Reserve (FCR), the automatic Frequency Restoration Reserve (aFRR), and the manual Frequency Restoration Reserve (mFRR) [36]. In grid operation, frequency responds to an imbalance between generation and consumption in the power system. For frequency stabilization, CR is provided as positive or negative CR to compensate for power imbalances and resulting frequency deviations [37]. The TSOs procure the CR products (control power and control energy) across control areas and partly cooperate with neighboring countries [38,39].

The prerequisite for participation in the CR market is the prequalification of the market participants with the respective connecting TSO [36]. The possibility of pooling makes it possible for small plants and loads to participate in the CR market. Through the prequalification procedure, potential suppliers ensure they meet the requirements for providing the various types of CR. Regarding technical competence, proper provision of the CR under operational conditions must be guaranteed [40].

When looking at other possibilities to market the electricity or flexibility of assets, another economic opportunity also considered in the interviews as a reference is to trade it in the short-term, physical electricity market. These markets include the day-ahead and intraday markets. This type of trading is referred to as direct marketing. Day-ahead trading refers to trading electricity for the following day, e.g., on the EPEX Spot in Paris or over-the-counter trading (OTC). Intraday electricity trading occurs on markets such as EPEX Spot and OTC trading [41]. It refers to the continuous buying and selling of electricity for same-day delivery. The intraday market is only served from Monday to Friday, while trading is possible on all days in the day-ahead market [42].

## 3 Research design and research methods

To deduce CSFs for future AS provision, we follow the DSR approach proposed by vom Brocke et al. [24] and Gregor and Hevner [23]. The DSR approach fosters the combination of practitioner experience and scientific literature knowledge to develop a design artifact in the form of our CSFs. According to Gregor and Hevner [23], our deduced CSFs contribute to a nascent design theory (level 2), which implicates " […] knowledge as operational principles/architecture” in the form of “constructs, methods, models, design principles, technological rules” [24, p. 342]. As we intend to create " […] new and innovative artifacts” ([43], p. 75) of elements necessary to achieve a goal [44,45], we consider CSFs as our research output. In our case, the goal can be defined as the future provision of AS in times of decentralization, decarbonization, and digitalization of the energy system. CSFs are " […] the limited number of areas where satisfactory results will ensure successful competitive performance for the individual, department or organization. CSFs are the few key areas where ‘things must go right’ for the business to flourish and for the manager's goals to be attained” [45, p. 84–85]. The CSF analysis is also applied in the energy sector to evaluate DER projects (e.g. Refs. [25,46,47]). In this context, a significant difference exists between CSFs, metrics, and key performance indicators (KPIs). In comparison to metrics, CSFs do not have to take the form of numerical measures. Unlike KPIs, CSFs do not have to be measurable [45]. In line with the DSR publication schema proposed by Gregor and Hevner [23], we conducted a five-step research design (see Table 1).

**Table 1:** Research design and applied methods.

DSR steps	*Problem identification*	*Gathering knowledge*	*Build design artifact*	*Evaluation*	*Communication*
**Steps and method**	**Step 1:** Literature review	**Step 2:** Expert interviews	**Step 3:** CSF analysis and qualitative	**Step 4:** Focus group discussion	**Step 5:** Development of a dodecahedron
**Tasks**	1.1 Define problem 1.2 Define literature search scope (keywords) 1.3 Conduct keyword-based literature search 1.4 Conduct backward, forward, and Google Scholar similarity search	2.1 Contact experts 2.2 Develop interview guidelines for TSO, PO, and DSO 2.3 Conduct and record interviews 2.4 Transcript interviews	3.1 Define category system for transcripts 3.2 Define coding guidelines and rules 3.3 Run-through transcripts 3.4 Deduce and formulate CSFs and CSF categories	4.1 Contact experts 4.2 Develop interview guideline 4.3 Evaluate CSFs according to comprehensibility, applicability, and completeness 4.4 Finalize CSFs	5.1 Choose the right framework to show relationships between CSFs 5.2 Identify relationships between CSFs 5.3 Present relationships in a dodecahedron
**Sources**	[[48], [49], [50], [51]]	[[52], [53], [54]]	[44,24,55]	[23,24]	[23,24]
**Output**	Relevant literature	Interview transcripts	CSFs	Final CSFs	Dodecahedron

The first step includes problem identification through a literature review in the introduction and the theoretical background. In this step, we followed the literature review guidelines [[48], [49], [50], [51]]. We concentrated on identifying critical factors influencing the successful provision of AS. Therefore, we searched for the following keywords: “ancillary service” OR “control reserve” OR “reserve market” OR “energy market” in the IEEE, Science Direct, AISeL, and SpringerLink databases. After screening the identified papers, we included 17 papers for further investigation. Based on this, we conducted a similarity search on Google Scholar and found 12 additional papers. Finally, this search process resulted in a total of 29 papers. In the second step, we intended to gather knowledge from expert interviews. Based on the literature review results (e.g. Refs. [2,7]), we formulated three interview guidelines (see Appendix) to flexibly address the different stakeholders - TSOs, POs, and DSOs - and their perspectives. As Wussow et al. [7] and Biedermann et al. [2] identified, technical, economic, and legal aspects as the main research fields for future AS provision, our interview questions focused on actions taken by grid operators, scheduling of control power types, market clearing, bidding and decision strategies (including sensitivity to high and low bids), and the process from power demand to power scheduling. According to Webster and Watson [48], the following concept matrix (Table 2) shows the process of selecting and formulating interview questions based on scientific literature.

**Table 2:** Concept matrix for the development of interview questionnaires.

Stakeholder	Interview questionnaire topics	References
TSO, PO	Process of AS provision	[2,26,27,31,[37], [38], [39]]
TSO, PO	Pricing	[7,17,33,35,37,[40], [41], [42]]
TSO, DSO	Competition (Competitors)	[[35], [36], [37], [38],^40^,^41^]
TSO, DSO, PO	Channel, signal for AS provision, communication to key partners	[13,16,25,33,34]
TSO, DSO, PO	Market-based provision of reactive power and other services	[2,7,35]
TSO, DSO, PO	Local and global AS provision	[9,13,21,30]
TSO, PO	Virtual power plants	[9,16,18,32]
DSO, PO	Information and communication technology (ICT) infrastructure, e.g., network topology, network resources	[8,11,12,15,28,34]
DSO	Static voltage maintenance	[2,7]
DSO, PO	Future market strategies, outlook	[3,8,11,12,15,28,34]
DSO	Integration of prosumers	[32]

Then, we conducted the expert interviews, which took place from October 2021 to August 2022 and lasted between 60 and 90 min. We contacted all known TSOs, DSOs, and POs in Germany. Unfortunately, only 13 experts (see Table 3) responded to our request. All interviews were fully recorded, transcribed, and anonymized. All interviewed persons provided informed consent for the publication of their anonymized statements. An approval by an ethics committee is not needed for this study because no clinical research, personal data, or case studies with human participants were conducted. The list of interviewed stakeholders is outlined in Table 3.

**Table 3:** Profiles of the interviewed stakeholders.

	ID	Job Description	Role/Company Description	Number of Employees
Interviews	Exp01	An analyst with a focus on aFFR	TSO	>2000
	Exp02	Product designer for CR products	TSO	>1000
	Exp03	Engineer in the grid control center for grid and system operation	TSO	>2000
	Exp04	Engineer in asset strategy	DSO	>1000
	Exp05	Consultant/engineer for grid planning	DSO	>1000
	Exp06	Head of a grid control system; senior management consultant	DSO	>250
	Exp07	Business field energy grids (asset management energy/development energy grids)	PO	>5000
	Exp08	EEG expert with a focus on biogas	PO	>1000
	Exp09	Product manager/energy for procurement	DSO	>100
	Exp10	Energy trader	DSO	<100
	Exp11	Energy trader	DSO	>1000
	Exp12	Energy trader	DSO	>1000
	Exp13	Energy trader	DSO	<100
Focus Group Discussion	Exp14	Researcher in energy engineering	University	–
	Exp15	Researcher in energy law	University	–
	Exp16	Researcher in network operation optimization	University	–
	Exp17	Researcher in power-hardware-in-the-loop experiments in DC systems	University	–

The third step includes the interview analysis using the open coding technique [56] to change the coding tags iteratively. After coding, we finally identified 45 categories, which we summarized into twelve CSFs. In the fourth step, we evaluated the comprehensibility, understandability, and usefulness of the CSFs with a focus group discussion, presenting the results and discussing them afterward [23]. In the fifth step, we developed a dodecahedron with twelve surfaces that present the twelve identified CSFs. We grouped the CSFs in an order that highlights their interdependence.

## 4 Results and findings

The interview statements were filtered and assigned as key statements to the respective CSFs. Finally, the most important statements are summarized in Table 4, and the corresponding experts are assigned. The CSF categories describe the associated CSF in more detail, referenced by the relevant expert.

**Table 4:** CSFs and its associated CSF categories.

CSF	Associated Category	Expert
Asset portfolio	Pumped and gas-fired storage power plants have the largest share in prequalified aFFR power.	1, 2
	There are few renewables in the portfolio for AS provision, as these are too risky regarding power reliability.	4, 5, 6, 7
	Renewables are particularly employed for intraday and day-ahead markets.	2, 3, 7, 8
Grid expansion	Expansion is done due to the weak and old infrastructure.	4, 6
	Expansion mainly in the form of larger transformers and lines.	3, 6
	Experimenting with the integration of more efficient standardized planning processes.	5
ICT transparency	No knowledge of the grid status in LV (and MV) for the DSOs.	4, 5, 9
	The highest interest is in more transparency for lower voltage levels.	5,6
	Ongoing installation of metering systems in transformers and households.	5,6
	Smart meter rollout causes many problems, e.g., technology acceptance and regulation.	6
ICT controllability and security	A plant's controllability, availability, and commitment are the main prerequisites for active participation and involvement in grid-stabilizing measures.	2, 3, 4, 5, 6, 7
	An ICT connection can only be established via analog wires and bus protocols and is set up via cascaded VPN zone communication.	3, 4, 7
	Customer acceptance is a major challenge with remote controllability of LV flexibilities.	5, 6, 7, 8
	Retrofitting plants for remote control technology is associated with high costs and effort.	2, 4, 5, 7
Communication	Unanimously striving for improved communication processes is necessary.	1, 2, 3, 5, 6, 7, 9
	Minor DSOs have to accept the decisions of superimposed/larger DSOs and fall in line.	5, 6, 9
	Remote plant controllability is an incentive to increasingly link the grid control systems to enable digitally automated communication.	1, 3, 5
	Grid operators express dissatisfaction with Redispatch 2.0: Barely any communication path to POs on how to proceed.	1, 5, 7, 8, 9
Control reserve	Four main criteria: Economic efficiency, operational background, remote controllability of the plant, and commitment.	1, 2, 3, 7
	The current demand for CR is sufficiently covered. An increase in demand with the expansion of DERs is expected.	1, 2, 3, 8
	Provision of CR is possible from all voltage levels.	1, 2, 3
	Control power and energy markets are add-ons. A preceding basic product is direct marketing.	2, 4, 5, 7, 8,9
Static voltage control	Fixed cosφ as a fundamental voltage control concept for DERs.	4, 5, 6
	Active control in lower voltage levels is mainly by transformers between HV and MV.	4, 6
	Continuous integration of innovative, voltage-controlling grid components due to high potential.	3, 4, 5
	Market-based procurement of reactive power in the lower voltage levels seems not viable due to the locality of providing reactive power.	3, 4, 5
Direct marketing: Intraday- and day-ahead trading	Direct marketing is the main venue for energy traders.	2,4, 5
	In the intraday market, short-term flexibility is balanced.	2
	The day-ahead market provides a certain level of guarantee and a low-risk level.	2, 6
Trading strategies	A bidding strategy has to fit the provider's investment portfolio and risk aversion.	10, 11, 12, 13
	Energy traders split their portfolios and offer them in various price categories, leading to different possible revenues at different risk levels and call probabilities.	3, 10,11
Integration of renewables	The duration of prequalification takes up to twelve months.	1, 2, 10
	Politics influence the economic viability, e.g., of BP.	8
	Projects to integrate renewables start up slowly.	3, 9
	Energy prices for renewables are not economically efficient.	6
Integration of prosumers & small plants, flexibility, and virtual power plants	Increase the need for transparency of LV level due to higher power demand and PV infeed.	5, 6
	Many open problems, e.g., aggregation, (de)central control, and economic efficiency.	5, 6, 9
	There must be no different treatment for aggregated pools.	8
Future strategies	The policy sets the portfolio guideline.	2, 4, 8, 9
	Grid expansion is indispensable, if necessary.	3, 4, 6, 7, 9
	Implementation of prioritization, coordination, and technology acceptance using LV flexibilities.	5, 6, 7, 8
	Digital transformation gains importance to automate processes at all voltage levels.	3, 5, 7, 8, 9
	The low potential of market-based reactive power provision.	3, 4, 5, 7, 9

### 4.1 Asset portfolio

The asset portfolio of a direct marketer usually consists of several plants, such as PV plants, BP, WPP (offshore and onshore), pumped storage power plants, and gas power plants. The current CR portfolio consists primarily of pumped storage and gas-fired power plants (Exp. 1, 2). It also includes batteries and BPs. Pumped storage power plants are an essential component of the CR due to their durability and large storage volume. In the asset portfolio of the future, offshore wind power plants will play an increasingly important role due to their more stable energy generation (Exp. 4, 5, 6, 7).

### 4.2 Grid expansion

Another identified CSF is grid expansion. Due to the increasing stress in the electrical distribution grid by DERs and a growing number of large residential demand components (EV and HP), there is consequently more stress on the grid components (Exp. 4, 6). This is mitigated by most of the DSOs with grid expansion. The local problems are attacked by integrating larger line radii and transformers. Due to the higher transmission capacity after the expansion, the thermal stress and the voltage drop over the line decrease (Exp. 3, 6).

Whereas this expansion is unavoidable in many grids because of weak line strength, the goal is to minimize grid expansion costs by sufficiently utilizing the existing grid components. DSOs are trying to integrate this strategy into their long-term grid planning by adapting their planning processes (Exp. 5).

### 4.3 ICT transparency

For DSOs to minimize their grid expansion, the stress within the grid needs to be known. As it currently stands, this is not the case, especially in the LV level (Exp. 4, 5, 9). Hence, the knowledge and transparency of the grid status implementing ICT is another CSF. There figures to be significant interest by DSOs to get more (real-time) information on the grid status, as ICT is implemented only in the HV level and some parts of the MV level, but there is still no transparency in the LV grid. ICT for remote control applications is beneficial for the short-term operation of the grid as it allows the DSOs to utilize the grid components to a greater extent with the knowledge of the current status (Exp. 5, 6). Planning processes are already possible as the grid data is present in a computational geographical information system for the DSOs. ICT integration is ongoing as more components like substation transformers are measured with ICT, and metering systems are implemented in households (Exp. 5, 6). Topics like the smart-meter rollout present problems for the DSOs because several processes and data must be integrated into their systems (Exp. 6).

### 4.4 ICT controllability and security

For active participation and integration in markets and the contribution to grid stabilization measures, it is a fundamental requirement that the plant is controllable and equipped with remote control technology. Two further criteria to be fulfilled are the commitment and reliable operation of the plant, which require good predictability (Exp. 2, 3, 4, 5, 6, 7).

The ICT connection can only be established via analog wires with, for example, 0–10 V signals and analog bus protocols and is set up via a cascaded virtual private network (VPN) zone communication as soon as third-party areas are entered. This approach ensures that Internet protocol addresses cannot be tracked. In addition, Internet connections are fully prohibited, so the VPN structures are not reached via the Internet but via multiprotocol label switching lines. The remote-control technology is provided to the computing centers via a specific and secure protocol, which is converted into another protocol to create a safety-related decoupling to the grid control system of TSOs. The chosen protocols vary in countries (Exp. 3, 4, 7).

Although most plants at the HV level and some large plants at the MV level are already connected to the remote-control technology, small plants from the MV and LV, on the other hand, are not. The remote-control capability for small systems at the LV level is also viewed critically since the integration of remote-control technology significantly increases the system's complexity, which requires an expanded range of expertise and qualifications of the installers in the construction and commissioning of small systems. A first approach to improve controllability and transparency at the LV level is sought through the smart meter rollout. This increases the potential of a reliable ICT infrastructure in the LV grid. The implemented interim model uses gateway administration to interface the smart meter and the grid control center. The gateway administration serves as the data collection pool. From there, the data is further communicated to the grid control center. The target model envisages direct access to the smart meter gateway in the long term. Concerning the provision of CRs, the information for the allocation after gate closure is provided via an application programming interface. Since the FCR is provided completely decentralized on a frequency-dependent basis, there is no data connection about the retrieval.

On the other hand, for the provision of aFFR, there is a redundant grid control connection between the pool and the grid control system of the TSO. The connection for the minute reserve is made via the merit order list server. In the long term, TSOs must be careful not to set the minimum product slice bids too low because the small product slices mean that data volumes increase enormously, leading to problems with process runtimes (Exp. 2, 4, 5, 7).

### 4.5 Communication

The statements regarding the communication between TSOs, DSOs, and POs are varied. 2/3 of the respondents communicate with the respective contact persons of the TSOs, DSOs, and POs. However, the communication paths are often still manual and not digitized. For example, information exchanges regarding maintenance or grid disruptions are often spread informally by telephone. Thus, plant controllability incentivizes digitally linking control systems to comply with high-security standards to enable automated communication processes. Although automated communication paths between TSOs and directly subordinate DSOs have existed in isolated cases until now, they are still far from mature. They are subject to significant expansion, a statement that all interviewees unanimously agree with and strive to improve. For example, the DSO is only slightly involved in the prequalification process to provide CR (Exp. 1, 2, 3, 5, 6, 7, 9). The TSO merely releases the requested prequalification power as the plant is connected to the distribution grid to avoid possible grid congestion. This process is again conducted manually and informally. A voltage reactive power management platform at the level of the first instance DSOs is a first step toward an automated process of reactive power demand distribution and regulating the responsibilities. The TSO analyzes the necessary demand and forwards it proportionally to the subordinate TSOs at the high-voltage level. This creates a cascaded process for meeting the requested demand (Exp. 1, 3, 5).

Regarding automation processes or new framework conditions for the grid operators, in many cases, the decisions of superordinate/larger DSOs must be accepted by the smaller DSOs. Smaller DSOs have little influence and fewer resources to participate in research approaches. Larger DSOs set the framework, which is often set based on project results (Exp. 5, 6, 9).

### 4.6 Control reserve

Nuclear power and coal phase-out have always been subordinate in providing CR. Therefore, the demand for CR is currently sufficiently covered. The expansion of DERs, especially e-mobility and HP, is expected to increase the power demand. The four TSOs coordinate the provision of CR in Germany and can be provided from all voltage levels. The responsible TSO's prequalification of the respective plant is required to participate in the CR markets, as explained in section 2 (Exp. 1, 2, 3, 8). The four main criteria and basic requirements a plant must fulfill to provide CR are economic efficiency, remote controllability, commitment (active management), and the operational background of the plant. Also, small plants can participate in the market by pooling offerings of several plants. For this purpose, it is necessary to prequalify the entire pool. The entire process for prequalification takes up to one year (Exp. 1, 2, 3, 7). The high requirements for participation in the CR market must not be underestimated, which means that the provision of CRs only serves as an additional product and is often subordinate to direct marketing. Accordingly, no plant is set up only to provide CR. Often, participation in the CR market fails due to the plant's high base costs, so it has to submit offers that are too high, which results in no or infrequent retrieval. Due to the low retrieval probability, it is not easy to operate the plant profitably (Exp. 2, 4, 5, 7, 8, 9).

The guiding principle of the TSOs is that despite the future internationalization of the markets, the CR requirement must be covered by the TSOs' resources and not based on imports.

Germany focuses on providing aFFR while retrievals in mFFR are relatively rare. In contrast, other countries, often smaller ones, relied more heavily on mFFR. Currently, the regular energy market has relatively low liquidity. Nevertheless, this market offers a higher potential for DERs to participate in CR provision because of the smaller time slice offers. The control power market, in contrast, does not offer much potential for DERs due to its high requirements and low commitment.

There is already a share of DER contributing to the CR market. Thus, most of the plants participating in the FCR market are covered by BSS. BSS has resulted in the FCR price in France being zero at times. In the FCR market, the allocation price is set at marginal cost. Since batteries have no marginal costs, this is efficient, but all other types of plants are pushed out of the FCR because it is no longer economically efficient for them. WPPs are mainly marketed at mFFR because the data transmission rate is not as high as at aFFR and FCR (Exp. 1, 2, 3).

### 4.7 Static voltage control

Regarding DSOs and grid stabilization measures, the focus is typically on local problems. Within these, voltage control and the market-based procurement (of reactive power) stands out as another CSF. Voltage control can also use flexibility and redispatch, grid components, and long-term planning of voltage control concepts (Exp. 3, 4, 5).

As plants' flexibility is not yet used for voltage control in the distribution grid, the DSOs concentrate on the voltage control concepts. Direct control of the voltage is mostly done by regulating the transformer between the HV and MV grid, as the MV and LV grids are rigidly coupled to a large extent. Nonetheless, the DSOs are aware of an increase in voltage problems due to changes in the MV and LV levels as they try to integrate more and more controllable local power transformers into their grids to allow the use of the full voltage band in all their voltage levels. Their relative share of all local transformers can be as high as 4 %, but it is rising as older transformers are substituted nowadays with a focus on the specific grid (Exp. 4, 6).

Regarding the third option – voltage control concepts – the DSOs typically specify a mandatory fixed cosφ according to the technical national connection guidelines in Germany. Other control concepts are considered for larger generation units of 30 kW or more, depending on the current grid status. The restriction for small plants on a voltage control concept that is probably not the best is made due to simplicity reasons for the installations of the plants (Exp. 4, 5, 6).

As a next step regarding the voltage control in the distribution grids, the market-based procurement of reactive power as a flexibility of the grid-connected plants has to be considered by the DSOs. However, due to the locality of the problem, there seems to be no possibility of a market-based procurement at the LV levels, as the risk of having an inefficient market or even no options outweighs the possibility of beneficial costs. Moreover, as the DSOs do not have a solution yet, the market-based procurement of reactive power is still of no interest to POs (Exp. 3, 4, 5).

### 4.8 Direct marketing: intraday- and day ahead trading

For most suppliers, direct marketing is the primary energy trading venue, while the marketing of CR is merely an add-on (Exp. 2, 4, 5). In the day-ahead market, long-term trading contracts can be concluded. In the intraday market, short-term energy surpluses or energy bottlenecks caused, for example, by short-term forecast errors, are offset. Thus, the volatility of energy is traded on the spot market (intraday and day ahead). According to the TSOs, the actual system balancing can be traded on the intraday market (Exp. 2).

Moreover, the underlying business is mostly the day-ahead market. The spot market has the least marketing risk due to the off-take guarantee. The small companies are more risk-averse and offer participation in the day-ahead market. Most POs also bid DERs in the spot market, as it is difficult to prequalify PV plants for CR because it cannot guarantee regulated output, and the forecasting accuracy is insufficient. DERs are rather scarce in the CR, so no efforts are made to build new plants for the CR (Exp. 2, 6).

### 4.9 Trading strategies

A key CSF is a suitable bidding strategy that fits the provider's investment portfolio and risk aversion. According to the experts, the day-ahead and the intraday market are the primary energy marketing venues. The day-ahead market, in particular, provides long-term planning security through guaranteed off-take.

Compared to the day-ahead market, the intraday market is riskier. According to DSOs, the long-term goal of the CR is to sell capacity above the mean value of the benchmark. Bidding strategies can be divided into standard, lucky, opportunistic, aggressive, and exit bids. Standard bids mean that the total capacity is offered on the CR market before offering on the day-ahead market. Initially, this happens at the normally expected power prices, which must be justified. The power left over, intended for the day-ahead market, is still offered on the CR market but at significantly higher prices. Working prices are based on the marginal costs of the plants (normally, working prices are offered simultaneously, and adjustments are always possible). Next are the lucky bids; these are the last power offerings at very high working or power prices, which are normally not marketable. Opportunistic bids mean that plants not connected to the grid are marketed at high prices in the aFFR market. According to DSOs, high working prices are over the benchmark prices. In the case of a surcharge, the plant is started; otherwise, the plant remains off. Another strategy is aggressive bidding, which is typically a strategy for big players. In this case, high prices are offered in the CR market. Failure to bid is no longer a major concern, as the opportunity of the control energy market continues to exist. The last strategy is the exit strategy, where extremely high control energy prices are offered to push oneself out of the merit order. A drawdown is considered when plants are broken or other markets are more lucrative (Exp. 3, 10, 11).

These strategies are distributed differently depending on how risk-averse and how large the trader's investment portfolio is. For example, risk-averse traders are more likely to offer most of the investment portfolio with the standard strategies in the regular reserve market and fewer investments in lucky and opportunistic bids, as the risk of not being awarded or called is too high. Big players with a more diversified and larger investment portfolio can put more shares into such riskier strategies (Exp. 10, 11, 12, 13).

### 4.10 Integration of renewables

This CSF describes the opportunities and challenges of integrating renewables into energy trading. Regarding the prequalification of renewables, wind turbines can be prequalified, but PV plants cannot yet be prequalified in the German AS market. Prequalification takes up to twelve months (Exp. 1, 2, 10). Due to the reduced fluctuation, offshore wind farms are much more attractive than onshore wind farms. Furthermore, offshore wind farms are newer, meaning they have a higher level of fine-grained ICT. In addition, it is not easy to integrate new plants into the regular reserve market. Most plants have existed for a long time, and new constructions are less planned (Exp. 3, 9). In addition, many POs experienced bankruptcies in the CR market when they bought a new plant and realized that the investment was not economically viable (Exp. 6). BPs are very much affected by the current political situation. They have great potential for aFFR, but if the regulatory environment changes, power generation from alternative energy sources such as BPs can be much more lucrative. There are also influencing factors in agricultural conditions, such as species protection. Other influencing factors are societal change (see Fridays for Future) and the hydrogen economy (Exp. 8).

### 4.11 Integration of prosumers & small plants, flexibility, and virtual power plants

As an integral part of grid components and grid expansion, the flexibility of systems is of great importance and, hence, another CSF regarding future AS in the distribution grid. In this regard, prosumer and their components EP, HP, PV systems, and BSS play a major role. The prosumers' changes are the main reason the DSOs are heavily interested in higher transparency and ICT integration into the LV levels (Exp. 5, 6). However, for the integration of their flexibility for AS purposes, a whole lot of stakeholders have to be considered: The households do have economic and comfort interests; the DSOs are interested in the stress of the local grid; the TSOs focus on the overall stability of the intercontinental grid, and market participants or traders care about revenues and liquidity. Additionally, a set of different questions has to be answered before prosumer flexibility can be used for grid purposes (Exp.5, 6, 9):

• Will the flexibility be controlled by a central control station of the grid operator or decentralized inside a transformer station?
• How can economic efficiency be reached for the investor and owner?
• How can small plants in the LV grid significantly impact the grid status?

The last question raises the problem of integrating the relatively small flexibility sets of many different plants, whose single flexibilities probably do not significantly impact. This makes aggregation of the plant indispensable.

In this context, VPPs play a major role and are of great interest to the grid operators and POs. They can allow more flexible use of the plants' power due to the formed association, but the topic also raises questions regarding the use and regulatory handling of aggregated plant parks. Due to discrimination, they must not be treated differently than other (conventional) energy plants, which sets very high standards. Nonetheless, grid operators and POs are optimistic about the potential contribution of LV plants and experience increasing attractiveness of the VPP concept (Exp. 8).

### 4.12 Future strategies

The derived future strategies refer to all the statements of the grid operators and POs and consequently thematically include aspects of the other CSFs. The focus of the interviews was on the short-to medium-term future of the operators. Consequently, not all perspectives were considered, e.g., the role of AS in cloud-based smart energy systems. Political objectives strongly drive grid- and plant operators' future actions and aspirations. In addition to the portfolio guidelines, these also set the investment focus of the grid and plant operators. The focus here is on economic efficiency and financial viability. Grid stabilization measures serve only as an additional product and are secondary to economic efficiency. In this context, an attempt is made to strive for grid-beneficial advantages with measures from the investment simultaneously. Other priorities set by political guidelines include digital transformation, grid and infrastructure expansion, and the hydrogen sector (Exp. 2, 4, 8, 9).

Progress is made steadily in terms of grid expansion, with thicker cross-sections installed and aluminum lines used to counteract potential voltage and electrical current problems. Large-scale conversions to controllable substations are preferred at the LV level, in addition to expanding transformer capacities. Large DSOs are already carrying out these conversions. Up to 75 % of conventional local substations will be adjustable. For the remaining 25 %, there will be no need for a controllable local power station. Following that, a retrofit and the grid expansion in the LV grid are preceded and prioritized by the plant complexity (Exp. 3, 4, 6, 7, 9). To improve transparency at the LV level, digital transformation gains importance to achieve process automation at all levels (Exp. 3, 5, 7, 8, 9). This will ultimately be accompanied by expanding communication paths between neighboring TSOs and DSOs and supporting the coupling of their grid control systems for full grid monitoring and transparency. Initial strategic approaches include using AS platforms between TSOs and DSOs and providing sub-aspects like a cascaded provision of reactive power (Exp. 5, 6, 7, 8).

## 5 Discussion, implications, and recommendations

To evaluate the CSFs with academic experts, we conducted a focus group discussion with four researchers in several research areas, see Table 3. The focus group discussion took place virtually and lasted 1 h. The authors moderated the focus group discussion and addressed the four main questions: comprehension, future research avenues, practical applicability, and missing aspects. The moderators explained to the four researchers (Exp. 14–17) the CSFs listed in Table 4. After the presentation of the CSFs, all researchers were interested in this study's results. The insights from the practical perspective in the different roles (TSO, DSO, and PO) were evaluated as contributions. Expert 14 stated that the CSFs' results open up new research areas that can be investigated in further work. The integration of DERs into the CR market and the transparency and acceptance of different voltage levels are currently not subject to the research focus of Experts 14 and 16. Considering LV flexibilities, Exp. 15 can use the insights from the CSFs analysis in daily academic research activities, e.g., simulations.

**Fig. 1:**
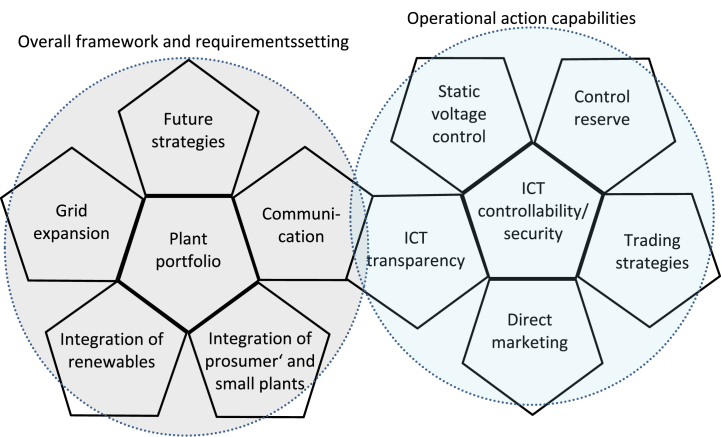
Connections between the CSFs are visualized as twelve faces mesh, resulting in a dodecahedron.

Twelve CSFs emerged from these interviews, divided into “overall framework and setting of requirements” and “operational action capabilities”. Therefore, we consider a dodecahedron a suitable representation to show the interactions between the CSFs because the dodecahedron is a platonic solid with twelve faces. These twelve faces can be folded together, which shows the connections between the faces, here CSFs. The unfolded mesh of the dodecahedron looks like two flower formations (see Fig. 1). These contain a central pentagonal plane connected at its edges with five other pentagonal planes. Each plane of the mesh represents a CSF. The identified CSFs are divided thematically into the “overall framework and requirements setting” (Fig. 1 left side) and the “operational action capabilities” (Fig. 1 right side).

The centers of the respective flowers set the foundation of the surrounding adjacent areas. Thus, the asset portfolio is the basic building block to determine the integration potential and exemplary grid expansion requirements. Plant controllability and security are the basic prerequisites to enable the operational participation of plants. The arrangement of the CSFs has been chosen according to the interactions between each other. Taking a broader view, it can be concluded that the influence of each grid operator also depends on the size of the company and its available funds and subsidies. The company's size is defined by the number of active employees, the size of its asset portfolio, and the size of its grid. Thus, larger grid operators are more involved in research and have a stronger influence on the design of future measures.

Furthermore, there is a close interaction between the CSFs future strategies, integration of prosumers and renewables, and direct marketing and trading strategies. In the future, prosumers and renewables will be better integrated into active grid operation to enable participation in the electricity markets, especially through flexibility in the LV grid. Approaches and solutions for hardware and software implementation are already available, but customer acceptance is still needed before such systems can be implemented. As seen in Fig. 1, communication and grid transparency are the links between the “overall framework and requirements setting” and “operational action capabilities”. Accordingly, the two flowers are mutually dependent. Without communication paths, the interaction between the roles of TSOs, DSOs, and POs across voltage levels is only possible to a very limited extent. However, grid transparency makes it possible to act in a way that serves the grid and the contribution of plants to grid stability.

To integrate DERs and especially prosumers for an active contribution to grid-serving measures, grid transparency and smart metering points are necessary to add remote controllability. Active participation is only possible if the plants disclose their status to the grid operator.

Concerning the correlation between static voltage control and grid expansion, we noted that the interviews confirm the studies [6] that there is already enough potential to provide static reactive power, e.g., regulated substations. The significant addition of DERs, especially EV and HP, will impact grid stability, congestion, and increasing demand for CR. Concerning the reactive power household, due to the continuously advanced grid expansion, the grid will also have sufficient potential to cover an increasing demand.

Due to the covered demands and existing potentials of static reactive power within the grids, the experts do not see any potential and need for an optimized market-based approach strategy. Furthermore, a market-based solution requires full remote controllability and increases plant requirements' complexity, rapidly reducing the efficiency and probability of market-based reactive power.

With a marketing and economic view, CR only serves as an additional product and is deliberately not linked to direct marketing (basic product). Therefore, CR is only provided as part of a marketing strategy. Consequently, the CSF ‘CR’ and the CSF’ direct marketing’ are only connected via the trading strategies CSF. According to the experts, direct marketing is the main market to generate profits in a long-term, schedulable, and riskless manner. If additional capacity is available in the asset portfolio, marketing on the CR market is considered. To participate in the CR market, many investments and administrative efforts (e.g., prequalification) must be completed, which can involve long periods. This effort must be weighed against the potential returns. Since participation in the CR market is influenced by many factors, the risk of losses is significantly higher, and the ability to forecast plant output is lower than in the day-ahead market.

Furthermore, significantly high profits can be achieved by utilizing trading strategies discussed in Section 4. However, this requires a high degree of risk appetite and know-how on the part of the traders. As a result, plants are not built for the sole purpose of providing the CR.

Many direct marketers, especially smaller companies, are overburdened with the new market conditions regarding personnel costs and the digital transformation of plants and systems. Following the restructuring of the market situation, the trading department must now be occupied 24 h a day, seven days a week. Previously, this was only 8 h per day. In addition, software solutions in the trading system must be updated according to the more fine-grained bidding system (e.g., algorithms for energy generation forecasting), and hardware components installed at the plants must be exchanged. As a result, many companies have withdrawn from the CR market until they are competitive again and can operate profitably; otherwise, their business will decline.

Considering the internationalization of the CR market and the resulting unification of the European market for CR, the experts see increased potential in the market for minute reserve capacity. In particular, systems with lower data transmission rates, such as older wind turbines, can participate in this market without requiring major hardware and software modifications.

The representation of the dodecahedron shows strong interdependencies of the CSFs. These factors influence the timeframe for achieving goals so that their implementation and achievement can be classified as short (S/1–3 years)-, medium (M/up to 10 years)- and long-term (L/more than ten years). Recommendations for further action can be derived from this to be able to identify thematic approaches for future research questions based on the knowledge gained from practical experience:

1) Political and regulatory requirements influence and control the actions of grid and plant operators (S).
2) Available budgets must be used efficiently for political aspirations:
  a. Economic and strategic action required from grid operators (M);
  b. Visions of grid operators are guided by the political goals (L).
3) Ongoing increases in DERs put growing stress on the electrical grid, resulting in a need for grid expansion that must be minimized by using flexibility (M).
4) Awareness and transparency of grid status through ICT is an advantage for short-term grid operation:
  a. Retrofit proves to be very complex due to many process adjustments and data integration procedures (M);
  b. Retrofitting on time involves a large cost factor. As a result, this recommendation is more likely to be achieved in the medium term (M);
  c. Full grid transparency at all levels improves operators' strategic and operational intervention. Effective process development is possible through using plants (L) efficiently.
5) Marketing in power and energy markets offers lots of opportunities but is also very challenging:
  a. CR is actually “only” an additional product because it requires much effort (S/M);
  b. POs rarely go directly to the market. Market participation often occurs via a third party, e.g., direct traders (S/M);
  c. Smaller players usually enter the market with low-risk and long-term contracts (S/M).
6) Complexity and tediousness in the prequalification process (M).
7) The digital transformation and automation of communication pathways, which amounts to both informal exchange and controllable processes, support the operational actions of the grid and POs and can lead to more efficient and secure operation of the electric power system (M).
8) Seeking more transparency at the LV level leads to the potential for prosumers to actively contribute to grid stability measures and for their flexibility to be utilized. However, the process toward full grid transparency at the LV level will be classified as medium-to long-term (L).

There are already many approaches to create more grid transparency and grid stability measures in the sense of processes and the provision of flexibility, but these are associated with great administrative, implementation, and cost efforts. Due to the complexity and interaction of all these factors, it is not realistic to establish these approaches on time. There is a lack of time, money, and competence.

## 6 Conclusions, limitations, and contributions

To address our RQ, we deduced CSFs for future AS provision in Germany by interviewing and analyzing the experience from practical active TSOs, DSOs, and POs. The focal points were selected to cover a comprehensive range of opinions, although it cannot be ruled out that other TSOs, DSOs, and POs who were not interviewed can have a different perspective. Only a snapshot of participants' opinions in the energy sector was shown, whereby care was taken to diversify the interviewees. The CSFs relate to the practical insights and experiences of TSOs, DSOs, and POs.

Based on the knowledge from scientific literature and experts, we deduced the twelve CSFs: Asset portfolio, grid expansion, ICT transparency, ICT controllability and security, communication, control reserve, static voltage control, direct marketing, trading strategies, integration of prosumers, integration of renewables, and future strategies. The CSF's interactions are visualized using the structure of a dodecahedron mesh and can be divided into “overall framework and requirement setting” as well as “operational action capabilities”. Our research provides detailed insights from the practice that cannot be found in the current literature, paving the way for further research opportunities and practical action. Recommendations for practical measures are drawn up, from which research gaps and needs can be extracted. This approach provides a behind-the-scenes look at how to solve technical research questions using social science methodology design. Hence, our research provides significant benefits to the stakeholders being described, who are thus enabled to identify the relevant CSFs for each role. Finally, we show that policy guidance drives the overall actions of TSOs, DSOs, and POs. Besides, our CSFs can be used as a checklist when developing novel research and practice projects. Further research can include the results in mathematical simulation analysis. The results can also be used to design and adjust economic and legal restrictions. Our CSFs show a way to consider specific CSFs in more detail, e.g., to implement behavioral patterns of TSOs, DSOs, and POs in existing algorithms. Based on the identified interrelationships and interactions between the individual CSFs, it can be conceivable to link existing individual approaches and thus develop larger overall systems, like research platforms. This can result in a co-running of different simulation areas, for example, the merging of technical and economic simulations.

## Data availability

Data will be made available on request.

## CRediT authorship contribution statement

**Jana Gerlach:** Writing – review & editing, Writing – original draft, Visualization, Validation, Resources, Project administration, Methodology, Investigation, Formal analysis, Data curation, Conceptualization. **Vanessa Beutel:** Writing – original draft, Resources, Investigation, Formal analysis, Conceptualization. **Carsten Wegkamp:** Writing – original draft, Resources, Investigation, Formal analysis. **Michael H. Breitner:** Supervision, Funding acquisition. **Stefan Geißendörfer:** Supervision, Funding acquisition. **Bernd Engel:** Supervision, Funding acquisition. **Karsten von Maydell:** Funding acquisition.

## Declaration of competing interest

The authors declare that they have no known competing financial interests or personal relationships that could have appeared to influence the work reported in this paper.

## Appendix

### Questionnaire for Transmission System Operators

1) What processes do you run for control power marketing?
  a) Which actors are involved?
  b) Solicitation of offers?
  c) Award of contract?
  d) Change in a strategy planned?
2) What factors influence pricing in balancing power markets?
  a) Time of day, day, weather, … ?
3) Roughly how many participants currently participate in the various balancing power markets?
  a) Are renewables already participating?
  b) Are there trends of certain types of plants succinctly serving a particular market?
  c) Are small offers rarely subsidized?
4) How does the call-off of the different types of control power occur?
  a) Signal of the surcharge?
  b) Signal for the time of provision?
5) How is communication generally done in system services with DSOs, POs?
  a) Are there plans for involvement or cooperation in the future marketing of system services?
  b) Do concepts already exist? (e.g., interactions of voltage maintenance/control power and congestion management).
6) How is it considered if the system service (esp. balancing power) is offered from a subordinate grid level?
  a) From which level, mainly?
7) Is it planned to procure reactive power at the market? (If yes, time horizon: short, medium, or long term).
8) To what extent does planning for marketing other system services (voltage maintenance, flexibility) already play a role for you?
9) In which market do you see the most potential in the long term? (Economically and from the point of view of grid stability)
10) Would you support or dispute the statement that providing system services (locally and globally) from low voltage makes sense or is possible?
11) What is your opinion on virtual power plants?

### Questionnaire for Distribution System Operators

1) What are long-term preparations being made at the DSO as part of network planning to consider voltage stability? (e.g., network topology, network resources).
2) What does your ICT infrastructure currently look like? What is foreseeable for the year 2030? If plants can be controlled: What signal controls the plant?
3) To what extent does static voltage maintenance currently play a role for you? (As a system service, short-term analysis and provision).
  a) How is the call-off done/is there a separate call-off process/is there a separate call-off process planned?
4) What future processes are already underway? (E.g., the need for market procurement of system services,
  a) Are market strategies planned?
  b) What could be the incentives for this?
  c) Are prequalification measures/requirements for market participation planned).
5) Are there any analyses or studies in which one can see after which events or seasonal influencing factors, increased voltage holding capacities, are required?
6) Are there any concepts already in place to integrate the procurement of voltage-holding measures? (e.g., market platforms and the interactions of voltage holding with control power/congestion management).
7) How is communication with TSOs generally done in the area of system services? For example, is notification given when system services are provided from distribution networks?
8) Are there plans to cooperate with TSOs or other DSOs in the future marketing or treatment of system services?
9) Would you support or dispute the statement that providing system services (local and global) from low voltage is reasonable or possible?
10) What is the likelihood that prosumers are expected to contribute?
  a) Are there specific types of plants where you prefer to participate in the market?
11) How local is the contribution to reactive power provision?
  a) Is it possible across voltage levels?
  b) As long as local allocation is given?
  c) Node-specific provision, circuit-specific?
12) Projects for future reactive power provision?
  a) Planned projects with industry partners are being pursued?
  b) Interest in further scientific exchange/contact partners from practice

### Questionnaire for Plant Operators

1) Which voltage levels do you serve with RE?
  a) What does your plant portfolio look like? In terms of the current 2021/2022 portfolio and, what will your 2030 asset portfolio look like? In which voltage levels?
  b) What does your ICT infrastructure currently look like? (ICT conditions/signal traces). What is foreseeable for the year 2030? Which signal will be used for plant control?
2) Concerning direct marketing, what do the process chains look like? (Offer submission to call-off). Which actors are involved?
  a) What is your strategy? (e.g., 80% of the plants are in the medium price segment and 20% “poker”, i.e., offer at high prices).
  b) Concrete offer and decision strategies in the day-ahead and intraday market?
3) Do you participate in the balancing power market or focus only on direct marketing?
  a) If yes: In which markets do you participate? From which voltage levels? With which plants?
  b) What does the process look like for participation in the balancing power market?
  c) Which players are involved? Bidding? Does ICT conditions/signal flow?
  d) If both markets are served, are there mixed strategies? (e.g., 20% balancing power and 80% direct marketing)
4) What does your ICT infrastructure currently look like? (Control power type-specific differences?) What is foreseeable for the year 2030? Which signal will be used for plant control?
  a) How is the retrieval of the different control power types done? Signal for allocation? Signal for provisioning time/call-off?
5) Outlook: In which market do you see the most potential in the long term? (Economic)
6) How do you communicate with the grid operators regarding the provision of system services?
7) To what extent do system services play a role for you? Is the marketing of other system services (voltage maintenance, congestion management/flexibility) being considered?
8) Is market-based reactive power provision an issue? (If yes, time horizon: short, medium or long term)
  a) Opinion on market-based reactive power procurement?
  b) Are (future) necessary processes for providing local system services (e.g., congestion management) already initiated/implemented?
9) Would you support or dispute the statement that providing system services (local and global) from low voltage is reasonable or possible?
10) What is your opinion on virtual power plants?
